# KYNURENINE METABOLITES INDUCE MICROGLIAL CELL SENESCENCE AND STIMULATE NEUROINFLAMMATION

**DOI:** 10.1093/geroni/igad104.3447

**Published:** 2023-12-21

**Authors:** Jimeace Bonaparte, Maxwell Cook, Steven Dixon, Cynthia Wright, William Hill, Gavin Wang

**Affiliations:** College of Charleston, Charleston, South Carolina, United States; Medical University of South Carolina, Charleston, South Carolina, United States; Medical University of South Carolina, Charleston, South Carolina, United States; Medical University of South Carolina, Charleston, South Carolina, United States; Medical University of South Carolina, Charleston, South Carolina, United States; Medical University of South Carolina, Charleston, South Carolina, United States

## Abstract

Aging is the greatest known risk factor for Alzheimer’s disease (AD) and other forms of dementia. Aging is associated with increased inflammation, which stimulates the activation of the kynurenine pathway and the production of kynurenine metabolites, including kynurenine (KYN), kynurenic acid (KYNA), 3-hydroxykynurenine (3HK), and quinolinic acid (QA). Previous studies have shown an elevated level of KYN and its metabolites in age-related diseases, including AD. Growing evidence suggests that age-associated accumulation of senescent cells plays a critical role in brain aging and AD pathogenesis. However, it remains to be determined how KYN or its metabolites affect neural cell senescence. The goal of this study was to investigate the effects of KYN and its metabolites on senescence induction in HMC3 human microglial cells. HMC3 cells were treated with a range of doses of KYN metabolites over multiple time points (3-48 hrs). Results showed that multiple independent senescent biomarkers were increased in 3HK-treated HMC3 cells, including SA-β-gal, whereas KYN-treated cells exhibited only a low level of SA-β-gal. The senescence induction effect of 3HK was further confirmed by increased expression of the p21 and p19 senescence markers. Furthermore, our data reveals that 3HK is the most potent metabolite to stimulate the secretion of the neuroinflammatory cytokine interleukin-6 (IL-6) by microglia compared to other KYN metabolites, and this result also demonstrates the SASP phenotype of 3HK-treated HMC3 cells. Taken together, these new findings suggest that KYN and its metabolites may contribute to the pathogenesis of AD via stimulating glial cell senescence and SASP-mediated neuroinflammation.

